# American Neurological Association

**Published:** 1881-09

**Authors:** 


ATLANTA
### Medical and Surgical Journal.

Vol. XIX.] SEPTEMBER—1881. [No. 6

### Reports of Societies.

### AMERICAN NEUROLOGICAL ASSOCIATION.

*Peculiar Effect of Bromide of Potassium in Insane Epileptics*—was the title of a paper sent by Dr. H. M. Bannister, of Chicago, and read by the Secretary.

The effect referred to by Dr. Bannister was the development of maniacal furor while the insane epileptic was taking bromide of potassium. A detailed history of one case was given in which the epileptic convulsions were arrested, but soon afterward maniacal furor developed, which subsided upon withdrawing the remedy, and returned with its renewal. Two other similar cases were cited.

Dr. Spitzka said that attention had been directed to this peculiar effect by a German alienist. He had also observed cases of the kind reported by Dr. Bannister, and believed that it would result in more than twenty-five per cent. of the inmates of our public institutions.

Dr. Jewell remarked that while the peculiar action of the bromide mentioned had not been unknown, it was important, whether the proportion of cases was as large as suggested by Dr. Spitzka, or was small. The fact should be recognized. He thought it probable that quite a number of epileptic insane, placed in asylums and kept upon the classical bromide treatment, were there as insane persons for that very reason. In all cases of epileptic insanity this possible effect produced by bromide of potassium should be constantly and distinctly born in mind; that is, one of the results of the bromide treatment of epileptic insanity, whether directly or indirectly was not determined, was maniacal furor, which rendered the person dangerous and liable to confinement and restraint.

Dr. J. C. Shaw had seen this peculiar effect more frequently in dispensary practice and among children than among the inmates of an asylum. He had seen one or two cases in the asylum of which he has charge, in which it became necessary to discontinue the drug.

Dr. Seguin had observed this peculiar condition in several cases, but he did not believe that it was due to the bromide. He thought that it was due rather to the suspension of the epilepsy. Vander Kolk had observed the same thing while treating epileptics with setons, and by allowing the patients to have fits the mental equilibrium had been restored. Dr. Seguin had observed the phenomenon following the suspension of the *petit* and the *grand mal*, and believed that it was due to the suspension of the epileptic condition rather than to the drug.

Dr. William A. Hammond, of New York, said that, while it was true the sudden suspension of epilepsy would produce mental aberration, he thought it was an infrequent occurrence when the frequency with which epileptic paroxysms were stopped without the development of these symptoms was considered. He had seen many cases in which acute mania had been produced with bromides, and he believed that there was a stage beyond which bromides could not be continued without producing mental aberration. The cumulative properties of the bromides should be studied more carefully than they had been. He had had four patients die while under the extensive use of bromides under his direction. Sometimes the dangerous symptoms could be removed by pergation.

Dr. Rockwell referred to a case in which, under his direction, the bromides had been continued eighteen months, with the view to carry the patient over, if possible, the point at which the convulsions had heretofore returned, and now, in view of Dr. Bannister's case and others reported, it became a question with him whether or not the remedy should be continued, as the patient was very much depressed, threatened suicide, etc.

Dr. Hammond said that if he had epilepsy he would take the bromides all his life.

Dr. A. D. Rockwell then reported, with remarks, a case of "Peripheral Paralysis from Pressure," which illustrated the fact that complete absence of electro-muscular contractility is not necessarily an unfavorable condition which cannot be removed. The point specially insisted upon was the persistent use of the galvanic current, although response might not be obtained for a long time. In the case reported one month elapsed before the muscles reacted at all, and in a case previously reported it was six weeks before galvanic contractility took place.

Dr. W. A. Hammond thought all would agree that it was more difficult to restore electro-muscular contractility where the paralysis was due to peripheral injury, than in a case in which it was of central origin.

Dr. Jewell thought that many cases of peripheral paralysis, in which there was evidence of absolute destruction of nerves, should be considered more hopeful than they usually were. He then cited a case in which the left lower extremity of a woman had been utterly useless for eleven months, with paralysis of sensation and motion after delivery with instruments, and by using the interrupted galvanic current, with massage, persistently for nearly a year, she was almost completely cured.

*Destructive Lesion of the Left Central Hemisphere*—Was the title of a paper sent by Dr. H. D. Schmidt, of New Orleans, and read by the Secretary.

The man received a blow from a club on the left side of his head in 1865 or 1866, and was unconscious for several weeks. There was also paralysis of the extremities of both sides. No scalp-wound was produced. The man recovered and retained all his mental faculties. At the autopsy a large hole—9½ ctm. horizontal by 5 ctm. vertical—was found in the bony vault, in the left parietal region, closed by a dense membrane. Beneath this was a large, ovoid hemorrhagic cyst. As the result of pressure produced by the cyst, the cerebral hemisphere presented a concave surface, the white substance of the brain had been destroyed, while the gray matter remained in the shape of a shell. The chief interest in the case centered in the extent of the lesions, and the length of time they had existed without without disturbing either the general health or the mental faculties of the patient.

Dr. J. S. Jewell, of Chicago, then read a paper *On the Eearly use of Strychnia in Myelitis*, the special object of which was to call attention to the early and free use of this remedy in acute and subacute affections of the special cord, in which its use had apparently been held to be unphysiological and even dangerous. The cases were those in which the accepted treatment had consisted in the use of ergot, massage, counter-irritation, electricity, etc, but in which, while the condition of the patient was daily and steadily growing worse, he had turned to strychnia in small and gradually increased doses, persistently used, and with the result of greatly benefiting the patient. In the persistent use of strychnia, exceeding care to prevent the patient from using the spinal cord (rest in bed perpetually, on the side or face), exceeding care to protect the patient from exposure (temperature of room maintained at 70° F.) so as to avoid a chill, were the important elements in his plan of treatment.

He had no theory to offer, except that the blood-vessels lost their *tonus* very quickly, and the vaso-motor apparatuses became fatigued and exhausted much earlier than was ordinarily supposed.

Dr. W. A. Hammond asked Dr. Jewell three questions:

First—Did the strychnia produce any tonic spasm?

Second—Were the cases cited uncomplicated ones of disease of the spinal cord?

Third—Might they not have been cases of spinal anæmia?

Dr. Jewell replied, first, that no tonic spasms were produced; second, that they were uncomplicated cases of disease of the spinal cord; and third, that he had not lost sight of the question of spinal anæmia, and had discussed it in the latter part of his paper, which had not been read.

Dr. E. C. Spitzka remarked that he would not dare to use the remedy in the early stages of inflammatory diseases of the cord. His experimental researches, however, had led him to the conclusion that the drug might perhaps be used in the early stages with benefit, if irritative symptoms were not present.

Dr. Hammond asked Dr. Spitzka if he did not discover, after giving excessive doses of strychnine to an animal, rupture of blood-vessdls in the substance of the spinal cord.

Dr. Spitzka said he always did when death was quickly produced.

Dr. Hammond asked if that fact did not indicate congestion of the spinal cord as an effect of the strychnia.

Dr. Spitzka said that he had always attributed the hemorrhages to respiratory spasm and death by asphyxia.

Dr. Hammond asked if, in a case of congestion of the spinal cord, the susceptibility of the cord to the influence of strychnia was not increased. He believed that Dr. Jewell's cases were anæmia, rather than congestion of the cord, and one strong point of evidence in favor of that position was the fact that in none of them were any of the physiological effects of strychnia produced, which must have occurred had the condition been that of congestion. In anæmia of the cord strychnia could be given in very large quantities without producing its physiological effect. He admired Dr. Jewell's judgment, respected in the highest degree his opinion, accepted without reserve his obserservations, but doubted the correctness of his diagnosis in the cases reported.

Dr. Jewell did not wish in the least degree to escape the criticism offered by such a matured judgment and experience as Dr. Hammond's, but, on the contrary, enjoyed them; for he was fully aware of the difficulties in making a differential diagnosis between spinal anæmia and spinal congestion, and was willing to meet them. In his judgment it was possible, in ninety-nine out of a hundred cases, to determine with tolerable accuracy whether we had to deal with decided spinal anæmia or decided spinal congestion—difficult as the cases were to distinguish the one from the other.

First—That acute and passive congestions of other organs of the body existed was well known and not disputed.

Second—That such congestions existed in the central nervous system (cranial) no one doubted.

By passave congestion he meant that which might be regarded as almost purely vaso-motor in its origin, and he spoke with special reference to arteries and capillaries. The quantity of blood in those vessels might be increased, in consequence of disorders of their walls, either muscular or vaso-motor. The condition was that of loss of *tonus* in the muscular coat of the blood-vessels, and the result, passive congestion.

Third—Spinal anæmia was made better by increased atmospheric pressure, whether it be obtained at the sea-level or in an air-tight chamber.

Fourth—Cases of passive congestion of the spinal cord improved when sent to higher altitudes.

Fifth—Spinal anæmia was made better by exposure to cold (cool, bracing climate), and increased in severity in warm climates.

Sixth—Spinal passive congestion, on the contrary, improved under the influence of warmth; hence, the patients did better in a warm than in a cold climate, and were beneficially affected by the local application of heat, the use of the Turkish bath, etc.

Seventh—There was a diminution in all the reflexes in passive congestion of the spinal cord.

By anæmia he meant contraction of a blood-vessel in consequence of a stimulus which reached it. That implied some excitation of the vaso-motor supply, which could be traced usually to some irritation, whether in the pelvic, the gastric or an intermediate zone.

Dr. Jewell continued his remarks at some length, and presented other points in the differential diagnosis.

Dr. Hammond thought it curious that Dr. Jewell saw cases of congestion in such numbers, and anæmia so frequently, when exactly the opposite would be true if Dr. Jewell's arguments concerning the effect of atmospheric pressure had any sure foundation. In other words, he regarded it as a fine illustration of transcendental philosophy to conclude that because a patient improved under such and such circumstances and was worse under opposite ones, he therefore must have congestion or anæmia, and it was begging the question completely.

Dr. E. C. Seguin was opposed to the doctrine of the existence of hyperæmia and anæmia, and looked upon the terms spinal hyperæmia and spinal anæmia as merely expressions for hypothetical views. He knew of no post-mortem or experimental evidence which supported the doctrines. All that had been heretofore adduced were observed and therapeutical phenomena put together in the right way to prove the existence of these two conditions. With respect to the spinal cord, researches had not shown any basis for the opinion that hyperæmia was the first step in myelitis. It was not even an important factor. The changes which took place were tissue-changes from first to last. With reference to the use of strychnia, however, he was inclined to agree with Dr. Jewell, but would suggest that the term "subacute myelitis" be changed to "subacute diffuse myelitis." He had administered the remedy in moderate doses in diffuse myelitis, following diphtheria, etc., with the best results, apparently due to the strychnia, and he was prepared to continue its use in acute diffuse myelitis.

The discussion was continued by Drs. Landon C. Gray and E. C. Spitzka and closed by Dr. Jewell, who said that he did not at all regard congestion as the first condition in inflammation, but simply as a clinical factor. Long before the congestion appeared, tissue-changes existed, and the hyperæmia was entirely secondary. Besides he did not share in that scientific skeptical nihilism presented by Dr. Seguin, which accepted nothing except what could be seen or smelled, or was susceptible of mechanical demonstration. It was all well in its legitimate field, but there was a *logical inference* which should be respected in all successful efforts to obtain the truth. That he had succeeded in obscuring Dr. Hammond's comprehension was more than he expected at the outset, but he promised to elaborate and set his views forth so clearly in the subsequent publication of the paper that even his learned opponent would be forced to acknowledge their value.

*How to use the Bromides*—Was the title of a paper read by Dr. Geo. M. Beard, in which he gave the following propositions:

First—The object, generally, in using the bromides was to produce a definite effect—bromization in a greater or less degree.

In a certain sense it was a disease artificially produced. Severe effects were not required. Mild effects of the remedy were all that were needed in most cases.

Second—To induce bromization it was usually advantageous to give immense doses. He often prescribed as high as a drachm or more. Doses of fifteen or twenty grains might be taken for some time without giving rise to symptoms of any kind. In some cases one hundred grains taken in one or two tumblers of water was all that would be required to break up an attack of hysteria, headache or prevent sea-sickness.

Third—They should be given in immense doses only for a short time, save in epilepsy and epieleptoid conditions. The cumulative effect must be recognized and constantly borne in mind. Sometimes a single large dose was sufficient to produce bromization, and that might occur within twenty minutes.

Fourth—Bromides, if used long and frequently upon any one patient, should be given in alternation or combination with tonics of some kind. Bromides one week and tonics the next, was a good rule.

One of the best remedies against bromization was powdered citrate of caffein, in from three to five grain doses.

Fifth—It was of advantage to use a number of bromides in combination.

While administering the bromides the patient should be kept under observation continuously.

The paper was discussed by Drs. W. A. Hammond, L. C. Gray, E. C. Seguin and J. S. Jewell.

Dr. Hammond preferred the bromide of sodium, if any of the salts were to be employed, but of late he had been using pure bromine, and had thus avoided the acne and the ulcerative manifestations of the bromide salts. His formula was

℞—Bromine, - - - - - - - ℈j
Aquæ, - - - - - - - ℥viij

M. S.—Teaspoonful well diluted with water.

Dr. Jewell had used pure bromine with favorable results in cases in which the alkaline bases could not be tolerated, and Dr. Seguin had reached the conclusion that the efficient agent was the bromine, and not the potassium.

*Nerve-Stretching in Locomotor Ataxy*—Was the title of a paper read by Dr. W. A Hammond, of New York.

The sciatic nerve was stretched. His conclusions were there was ground for hope with reference to the operation. A moderate extension, not to exceed an inch, was all that was necessary. The best place to reach the nerve was just where it came from under the biceps muscle. Too much stretching was bad. A *resume* of nine cases was given, seven by other observers and two of his own.

Dr. Jewell mentioned two cases reported recently by Bernhardt, in which the success had been marked, but not complete.

Dr. G. Hammond referred to a case in which the operation was followed by an aggravation of all the symptoms.

The discussion was continued by Drs. Rockwell, Morton, Spitsza, Amidon, Seguin and Birdsall, and a doubt concerning the propriety of the operation seemed to be the result; also a doubt whether the good results which had been obtained were due to the nerve-stretching or to the operation itself.—*New York Medical Record.*

